# Australia's Dengue Risk Driven by Human Adaptation to Climate Change

**DOI:** 10.1371/journal.pntd.0000429

**Published:** 2009-05-05

**Authors:** Nigel W. Beebe, Robert D. Cooper, Pipi Mottram, Anthony W. Sweeney

**Affiliations:** 1 School of Biological Sciences, University of Queensland, St Lucia, Queensland, Australia; 2 CSIRO Entomology, Long Pocket Laboratories, Indooroopilly, Queensland, Australia; 3 Australian Army Malaria Institute, Gallipoli Barracks, Enoggera, Queensland, Australia; 4 Communicable Diseases Branch, Queensland Health, Brisbane, Queensland, Australia; Asia-Pacific Institute for Tropical Medicine and Infectious Diseases, United States of America

## Abstract

**Background:**

The reduced rainfall in southeast Australia has placed this region's urban and rural communities on escalating water restrictions, with anthropogenic climate change forecasts suggesting that this drying trend will continue. To mitigate the stress this may place on domestic water supply, governments have encouraged the installation of large domestic water tanks in towns and cities throughout this region. These prospective stable mosquito larval sites create the possibility of the reintroduction of *Ae. aegypti* from Queensland, where it remains endemic, back into New South Wales and other populated centres in Australia, along with the associated emerging and re-emerging dengue risk if the virus was to be introduced.

**Methodology/Principal Findings:**

Having collated the known distribution of *Ae. aegypti* in Australia, we built distributional models using a genetic algorithm to project *Ae. aegypti*'s distribution under today's climate and under climate change scenarios for 2030 and 2050 and compared the outputs to published theoretical temperature limits. Incongruence identified between the models and theoretical temperature limits highlighted the difficulty of using point occurrence data to study a species whose distribution is mediated more by human activity than by climate. Synthesis of this data with dengue transmission climate limits in Australia derived from historical dengue epidemics suggested that a proliferation of domestic water storage tanks in Australia could result in another range expansion of *Ae. aegypti* which would present a risk of dengue transmission in most major cities during their warm summer months.

**Conclusions/Significance:**

In the debate of the role climate change will play in the future range of dengue in Australia, we conclude that the increased risk of an *Ae. aegypti* range expansion in Australia would be due not directly to climate change but rather to human adaptation to the current and forecasted regional drying through the installation of large domestic water storing containers. The expansion of this efficient dengue vector presents both an emerging and re-emerging disease risk to Australia. Therefore, if the installation and maintenance of domestic water storage tanks is not tightly controlled, *Ae. aegypti* could expand its range again and cohabit with the majority of Australia's population, presenting a high potential dengue transmission risk during our warm summers.

## Author Summary

Current and projected rainfall reduction in southeast Australia has seen the installation of large numbers of government-subsidised and ad hoc domestic water storage containers that could create the possibility of the mosquito *Ae. aegypti* expanding out of Queensland into southern Australian's urban regions. By assessing the past and current distribution of *Ae. aegypti* in Australia, we construct distributional models for this dengue vector for our current climate and projected climates for 2030 and 2050. The resulting mosquito distribution maps are compared to published theoretical temperature limits for *Ae. aegypti* and some differences are identified. Nonetheless, synthesising our mosquito distribution maps with dengue transmission climate limits derived from historical dengue epidemics in Australia suggests that the current proliferation of domestic water storage tanks could easily result in another range expansion of *Ae. aegypti* along with the associated dengue risk were the virus to be introduced.

## Introduction

*Aedes* (*Stegomyia*) *aegypti* (Linneaus) is an important vector of dengue and other arboviruses. Despite its limited flight dispersal capability [1],[2], its close association with humans and its desiccation-resistant eggs have facilitated many long distance dispersal events within and between continents, allowing it to expand its range globally from its origin in Africa. Its global emergence and resurgence can be attributed to factors including urbanisation, transportation, changes in human movement, and behaviour, resulting in dengue running second to malaria in terms of human morbidity and mortality [3],[4]. Global historical collections and laboratory experiments on this well studied vector have suggested its distribution is limited by the 10°C winter isotherm [5], while a more recent and complex stochastic population dynamics model analysis suggests the temperature's limiting value to be more towards the 15°C yearly isotherm [6].

**Figure 1:**
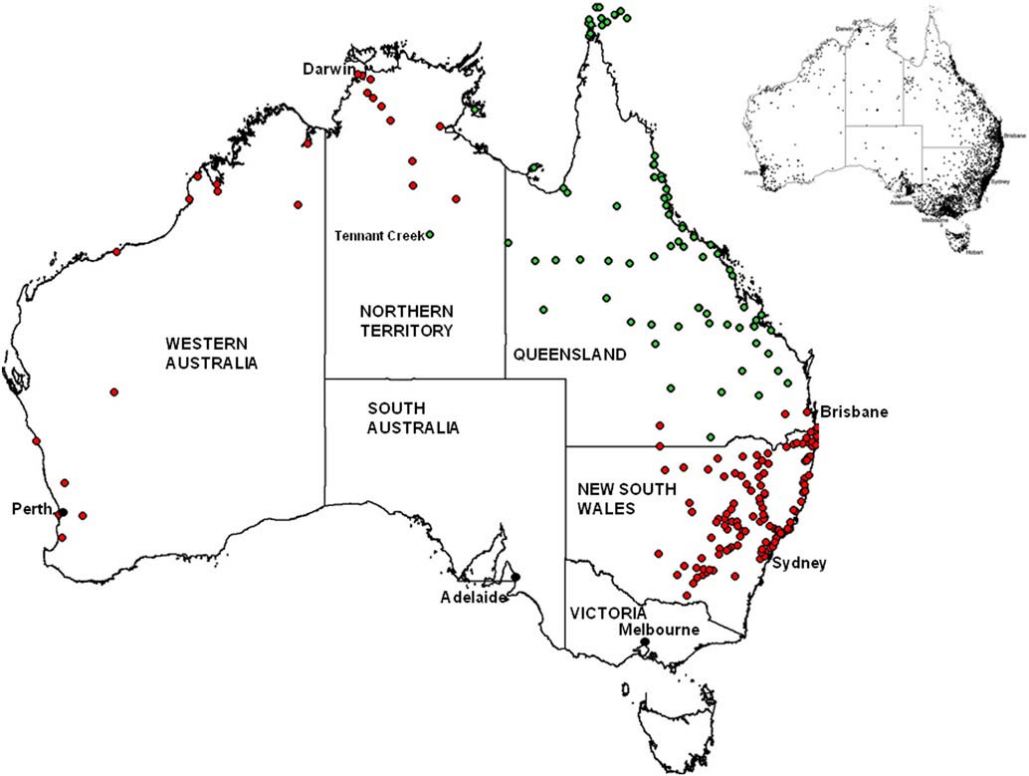
Map of Australia showing the 234 *Ae. aegypti* collection sites described in Table S1. Almost all localities (except site 219 and 220) can be regarded as historical collections while red sites indicate historical sites where *Ae. aegypti* is no longer found and green sites are regarded as contemporary sites, collected since 1980. Top right map displays the current Australia resident population distribution and each dot represents approximately 1000 people (Source: Australian Demographic Statistics (3101.1)).

While historical surveys in Australia have indicated that *Ae. aegypti* occurred over much of the continent (see Fig. 1), its range has receded from Western Australia, the Northern Territory and New South Wales (NSW) over the last 50 years. It is now only found in Queensland [7],[8], although recent incursions into the Northern Territory have required costly eradication strategies [8]. The significant reduction in vector distribution has been attributed to a combination of events including the introduction of reticulated water, which reduced the domestic water storage requirements of households that had provided stable larval sites [7],[9], as well as the removal of the railway-based water storage containers hypothesised as being responsible for the long distance dispersal events of *Ae. aegypti* into rural regions in NSW via steam trains [7],[10].

Today, epidemic dengue is limited to regions of Queensland where *Ae. aegypti* is extant, and the frequency of outbreaks has increased constantly over the past decade [11]. Historically, epidemics of dengue were recorded in northern Queensland in the late 1800s and in southeast Queensland in 1904–05 [10]. Dengue epidemics in 1926, 1942 and 1943 all extended from Queensland south into NSW, stopping only on the arrival of winter [12]. Derrick and Bicks [12] found that these dengue epidemics ceased when the outside temperature reached a wet bulb isotherm of between 14–15°C and suggested that a parameter of 14.2°C mean annual wet bulb isotherm (T_W_) best represented the limiting parameter for the 1926 epidemic.

The current drying of southeast Australia has placed this region's urban and rural communities on escalating water restrictions, with anthropogenic climate change forecasts suggesting that this drying trend will continue [13]. To mitigate against this regional drying effect and the stress it places on domestic water supply, state government rebate programs have been initiated to encourage the installation of large (>3000 L) domestic water tanks in towns and cities throughout this region. Data from the Australian Bureau of Statistics [14] records that in 2006, 20.6% of all Australian household dwellings had rainwater tanks.

Given the expansion of domestic rainwater tanks in southern Australia, and assuming these domestic water tanks can provide oviposition sites, we ask this question: can climate be assessed to determine the distributional limits of *Ae. aegypti* and dengue in Australia? We first use a genetic algorithm to develop ecological niche models for the distribution of *Ae. aegypti* in Australia (using data points drawn from both historical and contemporary collection sites) and evaluate the potential distributional limits of *Ae. aegypti* in Australia under today's climate and in future projected climate change scenarios. We map these limits in relation to published experimental and theoretical projections of *Ae. aegypti*'s temperature limits and then compare all projections to dengue transmission climate limits obtained from epidemiological studies of historical dengue epidemics in Australian. We find that human adaptation to climate change – through the installation of large stable water storage tanks – may pose a more substantial risk to the Australian population than do the direct effects of climate change. Additionally, we find that using point occurrence data and environmental parameters of climate and elevation to map the distribution of *Ae. aegypti* in Australia prove deceptive and require interpretation as some *Ae. aegypti* collection sites exist outside our ecological niche models and both theoretical cold temperature limits. This suggests that *Ae. aegypti's* domestic behaviour – with a lifecycle based around human habitation that includes blood-feeding and resting indoors as well as egg laying in artificial containers around houses – plays an influencing role on distribution.

## Materials and Methods

### Distribution of *Aedes aegypti* in Australia

Coordinates for a total of 234 *Ae. aegypti* collections sites are described in Table S1. Historical collection sites were compiled [7],[9],[15],[16]. Contemporary collection sites were regarded as those collected since 1980 because most country towns had moved to reticulated water, steam powered trains had been replaced by diesel, and the common railway station water-filled fire buckets were removed [9],[17],[18]. Contemporary sites also include collections made between 1990 and 2005 from southeast Queensland (P. Mottram, unpub. data), and the Northern Territory (P. Whelan, unpub. data).

### Base climate layers

Raster ASCII grids were generated for Australia at a spatial resolution of 0.025° (approximately 2.5 km) for eight climate variables plus elevation. These included annual mean rainfall and annual mean temperature produced by BIOCLIM using the ANUCLIM software package [19] as well as mean values of maximum temperatures and minimum temperatures for the months of January and July produced by the ESOCLIM component of ANUCLIM. This procedure involved the use of monthly mean climate surface coefficients, generated by the thin plate smoothing spline technique ANUSPLIN [20] from Australian Bureau of Meteorology climate data between 1921 and 1995 [21]. The geographic coordinates of the meteorological stations were used as independent spline variables together with a 0.025° digital elevation model (DEM) for Australia generated with ANUDEM [22] which acted as a third independent variable. As atmospheric moisture is known to be an important factor in terms of the survival and longevity of adult mosquitoes, mean values of dewpoint for January and July were generated with ESOCLIM to provide this.

### Climate change layers

A further series of ASCII grids were generated from climate change scenarios using OzClim version 2 software [23],[24] at a spatial resolution of 0.25° (approximately 25 km). The scenarios used for this study were for 2030 and 2050 using CSIRO: Mk2 Climate Change Pattern with SRES Marker Scenario A1B and mid climate sensitivity. The output variables corresponded to the predicted change from the base climate for the rainfall and temperature parameters generated with ANUCLIM.

This version of OzClim outputs vapour pressure rather than dewpoint as a measure of atmospheric moisture. For the present study vapour pressure grids for the predicted change from base climate for January and July were generated and the grid cell values were converted to dewpoint by applying the inverse of Tetens' equation [dp = (241.88×ln(vp/610.78))/(17.558−ln(vp/610.78)]. This mathematical procedure was implemented with the use of ImageJ software (publicly available at http://rsbweb.info.nih.gov/ij) together with the raster operations of TNTmips (MicroImages Inc., Lincoln, Nebraska).

The environmental layers used for climate change modelling were prepared by resampling the OzClim outputs to the geographical extents and grid cell size of the ANUCLIM grids using TNTmips. The resampled outputs were then added to the corresponding ANUCLIM base climate layers to produce the environmental layers predicted for the chosen climate change scenarios.

### Ecological niche modelling

DesktopGarp version 1_1_6 [25] was used for ecological niche modelling in a manner similar to our earlier studies [26]. Models derived from the historical climate data were generated using the record sites for *Ae. aegypti* as inputs together with the eight base climate layers and elevation (the ANUDEM generated DEM is described above) to model the range of *Ae. aegypti*. Species record sites and the climate change layers for 8 environmental parameters were derived from the climate change scenarios for 2030 and 2050 as well as the elevation layer. We utilized the medium sensitivity which corresponds to a global warming of 2.6°C for a doubling of CO_2_ from 280 ppm to 560 ppm [27]. The GARP procedure was implemented using half of the species record sites as a training data set for model building and the other half for model testing. Optimization parameters included 100 models for each run with 1000 iterations per model and 0.01 convergence limits. The best subsets procedure [28] was used to select 5 models which were added together using TNTmips to produce predicted range maps for each species.

### Theoretical temperature limits for *Ae. aegypti* extrapolated across Australia

Previous studies of the distributional limits of *Ae. aegypti* were used to develop distribution maps for Australia. Christophers [5] hypothesised a climate limit of 10°C winter isotherm based on historical global collection data and laboratory-based experiments. We also evaluated the hypothetical limit from Otero and colleagues [6], who used a complex stochastic population model that incorporates the lifecycle parameters of *Ae. aegypti* to suggest a 15°C annual mean isotherm. Both these values were incorporated into distributional maps of Australia using TNTmips.

### Climate limit of dengue transmission in Australia

Dengue transmission maps were developed using data from historical dengue outbreaks in Australia [12]. This work found that these dengue epidemics ceased when the outside temperature reached 14–15°C wet bulb isotherm and that a single parameter of 14.2°C annual mean wet bulb isotherm (T_W_) best approximated the limit of the 1926 epidemic – probably as a result of reducing the mosquitoes' feeding activity and the ability of the virus to replicate. This 14.2°C annual mean wet bulb isotherm value was mapped onto Australia for the current climate using TNTmips and three seasonal increments: the annual mean, the warmest quarter (December–February), and the coolest quarter (June–August).

## Results

### Distributional projections of *Ae. aegypti*: GARP modelling

**Figure 2:**
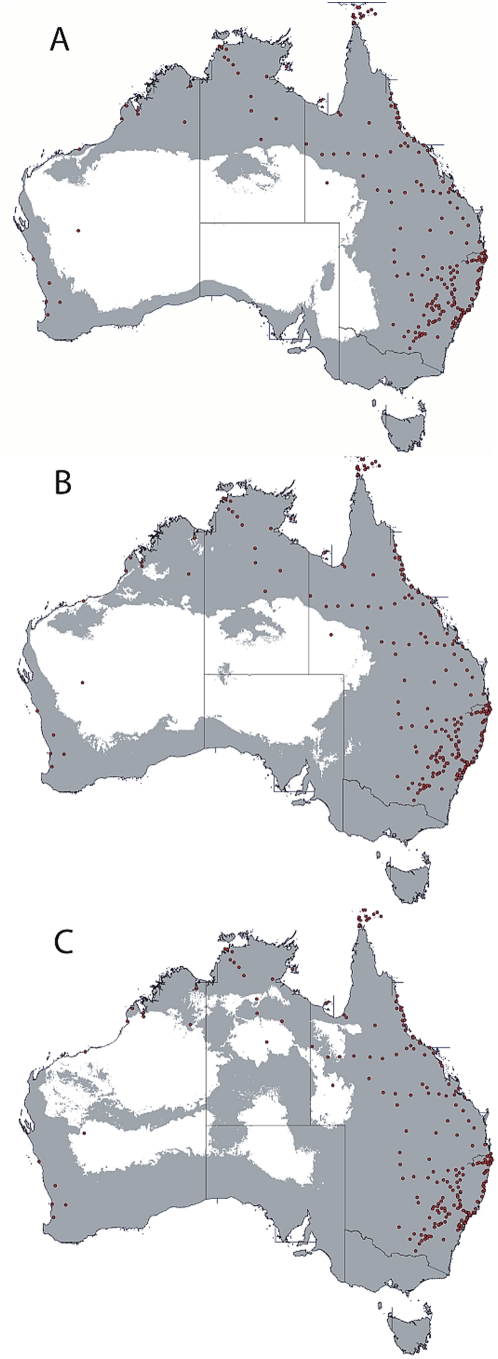
Distributional projections of *Ae. aegypti* in Australia based on 234 collection sites and built using desktop GARP and eight climatic variables. Panel A is the base layer projection (gray region) for the climate of 1995 and is regarded as current climate. Panel B is the projection of the forecasted climate changes for 2030 mid scenario. Panel C is the projection of the forecasted climate changes for 2050 mid scenario.

Distribution sites for *Ae. aegypti* in Australia (234 sites) were collated and displayed in a single map using GPS coordinates (Table S1 and Fig. 1). Ecological niche models were built with desktop GARP to produce a best subset model that showed agreement with the full complement of *Ae. aegypti* collections in Australia (Fig. 2A). In this projection, much of northern, eastern and southeast Australia was projected to present a suitable niche. This model closely tracks an annual rainfall pattern of less than 300 mm. However, the excluded region around central Australia included two *Ae. aegypti* positive collection sites (Meekatharra in central Western Australia and Boulia in Queensland): both collection localities are small regional centres on main inland transport routes.

The projected climate change scenario for 2030 produced distributional models with small expansions of the base model envelope, mostly evident in southern Australia (Fig. 2B). Likewise the 2050 model (Fig. 2C) extended the 2030 trend, resulting in a reduced niche in north-west Australia's Pilbara region while parts of central Australia opened up as a potential niche.

### Theoretical temperature limits of *Ae. aegypti*

**Figure 3:**
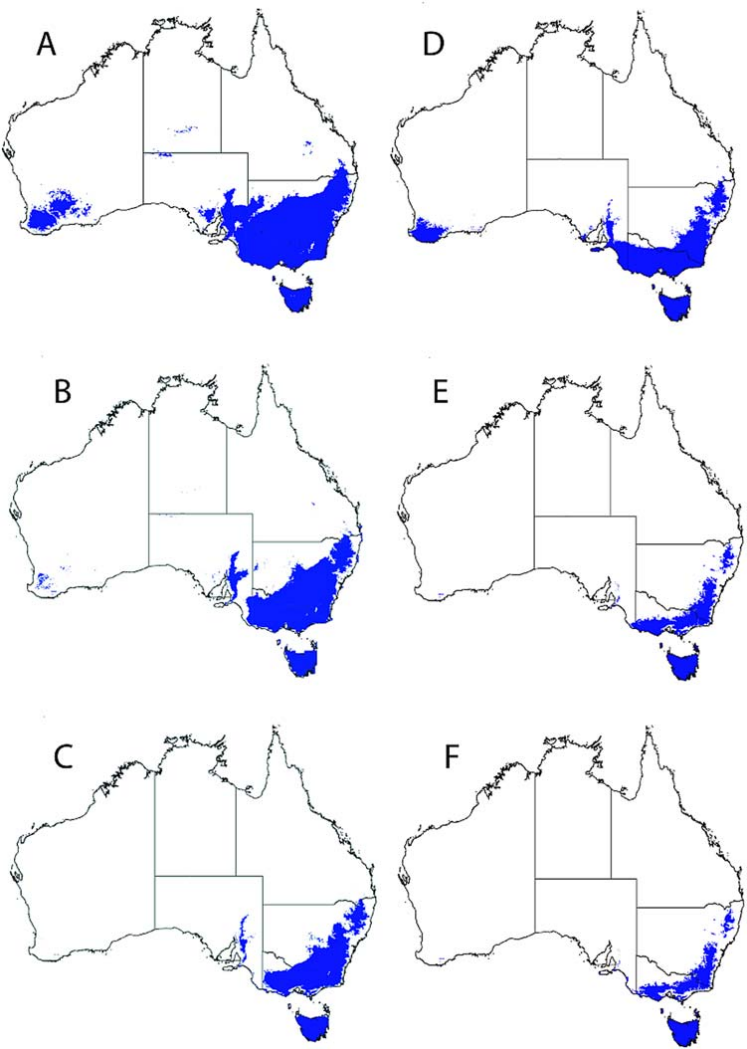
Theoretical distribution limits for *Ae. aegypti* and dengue transmission in Australia. Panels A–C represent the 10°C July isotherm with panel A the base layer projection for the current climate (1995). Panels B and C show the 10°C July isotherm limit of the climate change (mid) scenarios for 2030 and 2050 respectively. Panels D–F show distribution limits of *Ae. aegypti* in Australia based on the climate limit of 15°C annual mean isotherm. Panel D is the current climate (1995), panels E and F show the 15°C annual mean isotherm for climate change mid scenarios 2030 and 2050 respectively.

The temperature limit parameters of 10°C winter isotherm [5] and 15°C annual isotherm [6] were used to build theoretical isotherm limits for *Ae. aegypti* in Australia (Fig. 3). Figure 3A shows a 10°C winter isotherm limit base map for the current climate and OzClim projections were then generated for 2030 and 2050 by adding the projected changes to this base map (3B and 3C respectively). The 15°C annual isotherm limits were similarly generated using a base map and adding the OzClim changes. Both the 10°C (average winter) and 15°C (average annual) limits incorporate the major state capitals cities – Brisbane, Sydney, Adelaide and Perth. When these isotherm limits were subjected to the climate change scenarios for 2030 and 2050, the projection expanded to include the other mainland state capital, Melbourne (Fig. 3B and 3C).

Several *Ae. aegypti* collection sites occurred well within the two theoretical cold climate limits. Table 1 details six *Ae. aegypti* collection sites as examples where the annual mean temperature and the mean temperature for July (calculated as (mintemp+maxtemp)/2) fall below the theoretical values and range from 12.4–15.4°C and 5.2–7.6°C respectively.

**Table 1:** Collection sites in NSW that fall below theoretical cool temperature limits.

Site	Locality	Annual mean temp (°C)	Max/min temp July (°C)	Mean temp July (°C)	Elevation (M)
98	Breadalbane	12.4	10.3/0.2	5.25	701
116	Culcairn	14.7	11.9/2.2	7.05	221
187	Wagga Wagga	15.4	12.8/2.4	7.6	177
133	Junee	15.1	12.4/2.2	7.3	295
131	Harden	14.3	12.1/1.2	6.65	396
189	Wallendbeen	13.9	11.6/1.0	6.3	468

### Theoretical dengue transmission limits

**Figure 4:**
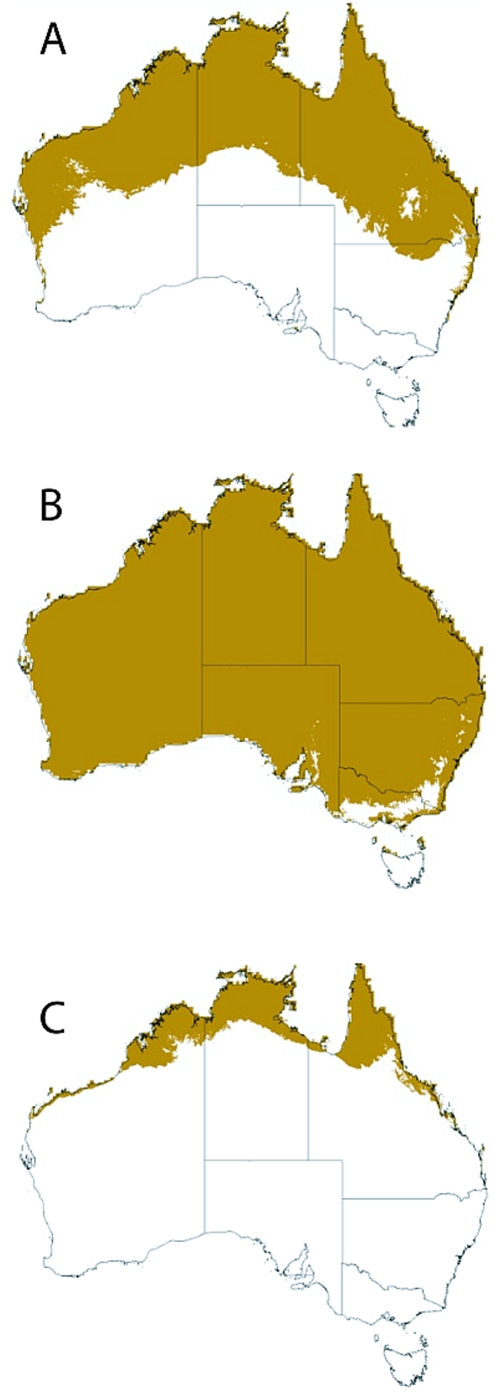
Employing a hypothetical dengue climate limit estimated from epidemics in Australia that stopped on the arrival of winter where the outside temperature fell to a wet bulb isotherm (T_W_) of 14–15°C [12], we mapped a 14.2°C T_W_ isotherm onto Australia using three temporal increments. Panel A represents the 14.2°C annual mean T_W_ for Australia [12]. Panel B represents the 14.2°C T_W_ for Australia's warmest quarter (December–February), representing summer transmission. Panel C represents the same isotherm for Australia's coolest quarter (June–August), representing potential year-round transmission.

Derrick and Bicks [12] suggested that dengue transmission stopped between the 15°C and 14°C T_W_ isotherm and suggested that a 14.2°C T_W_ annual mean isotherm best approximated the temperature limit for transmission in the 1926 dengue epidemic. We applied this isotherm to Australia for the annual mean isotherm (Fig. 4A) as well as the warmest quarter isotherm (summer; December–February, Fig. 4B) and the coldest quarter isotherm (winter; June–August, Fig. 4C). These climate limit maps indicate that if the vector could re-establish itself throughout its former range then much of northern tropical Australia would be receptive to dengue transmission year round and transmission would be possible throughout most of Australia during the summer months.

## Discussion

Can the historical distribution of *Ae. aegypti* in Australia provide an insight into the potential distribution potential of this mosquito? Using 234 different spatial data points generated from historical and contemporary collections of *Ae. aegypti* in Australia, we developed ecological niche models to hypothesise the potential range expansion of this mosquito under today's climate and under future climate change scenarios for 2030 and 2050 using OzClim mid sensitivity values that correspond to a global warming of 2.6°C for a doubling of CO_2_ from 280 ppm to 560 ppm [27]. In Australia general warming estimates are approximately 1.0°C by 2030 and 1.2 to 2.2°C by 2050, the latter values being dependent on CO_2_ emissions. While rainfall (outside of far north Australia) is estimated to decrease by 2% to 5%, southern Australia is projected to encounter a 5% reduction in rainfall [13]. Our GARP model for current climate suggested that *Ae. aegypti* could potentially coexist with over 95% of the Australian population and this distribution did not change significantly, with regard to the Australian population distribution, under either the 2030 and 2050 climate change scenarios.

Only the highly arid central Australian region was excluded from the projection (annual rainfall less than 300 mm). The GARP model did not show southern cold climate thermal limits in Australia, probably due to the presence of several *Ae. aegypti* collection sites from inland New South Wales that show cool climate parameters. We then mapped two theoretical cool climate limits across Australia – the 10°C winter (July) isotherm [5] and the 15°C annual mean isotherm [6]. Of these two isotherm limits the 15°C annual mean isotherm appeared more representative of the known distribution of *Ae. aegypti* in Australia, although collection sites did exist outside these temperature isotherm limits.

It remains unknown if the cold climate tolerant populations were breeding in the warmer months and surviving the colder winter months as eggs [29], or were surviving as larvae. With regard to these questions, observations have been recorded of viable *Ae. aegypti* larvae in ice encrusted water [5],[7], while experiments have suggested that a water temperature of 1.0°C can be lethal over 24 hours, but larvae can be viable at a constant 7.0°C for over a week [5]. At the other temperature extreme, laboratory experiments show that *Ae. aegypti* larvae perish when the water temperature exceeds 34°C while adults start to die off as the air temperature exceeds 40°C [5]. Domestic water tanks in Australia contain thousands of litres of water that would – in combination with the mosquitoes' domestic (indoor) nature – provide a buffer to temperature extremes and assist mosquito survival in what may appear unsuitable environments. For example, *Ae. aegypti* exists and transmit dengue in India's Thar desert townships in north-western Rajasthan, where the mosquito utilises household pitchers and underground cement water tanks. [30].

The incongruence between the temperature limits and our ecological niche models highlights the difficulties of using what are essentially sophisticated climate pattern matching procedures to study an organism with a biology and ecology strongly influenced by human activity. Fortunately, we can directly compare our GARP model with a new mechanistic model of the same organism over the same environment [31]. This mechanistic model utilises biophysical life processes parameters such as the effects of climate on reproduction and larval development. Larval development in both rainwater tanks and smaller containers were assessed and the potential distribution of *Ae. aegypti* was projected across Australia. Projections using rainwater tanks larval development resembled our GARP model for Northern and central Australia, but unlike our projections, a southern cold climate thermal limit was identified which was actually lower than the published parameters displayed in Fig. 3 [5],[6]. Apart from showing the clear advantage of a bottom-up approach for modelling this mosquito, this study supports the hypothesis that domestic rainwater tanks contributed for the historical southern distribution of *Ae.aegypti* in Australia.

Humans not only facilitate long distance dispersal events for this mosquito, co-habitation with humans can provide thermal buffers to the outdoor climate as adults rest indoors, and domestic rainwater tanks can provide stable oviposition sites. When the theoretical distributions (GARP models and temperature limits) and actual *Ae. aegypti* distributions are viewed alongside the expansion of domestic water tanks underway in Australia, a trend emerges where *Ae. aegypti* could potentially exist year-round in today's climate throughout the southern Australian mainland. This potential distribution includes the metropolitan areas of Brisbane (pop 1.8 million), Sydney (pop 4.2 million), Adelaide (pop 1.1 million) and Perth (pop 1.5 million). Additionally the climate change temperature limit projections for the mid scenario 2050 see this range expand to include Melbourne (pop 3.6 million). The addition of a theoretical dengue virus transmission limit parameter (we used a 14.2°C wet bulb isotherm) suggests an overlapping dengue risk in many of Australia's metropolitan regions during the summer months (December–February).

The potential for dengue virus introduction to these regions through travellers from endemic regions (including north Queensland) during summer presents a transmission risk that can be inferred by the current incidence of imported and endemic cases of dengue in Australia – many of which enter Australia through national and international transport nodes. For example, for the year to June 2008 there were 250 dengue notifications for Australia, of which 113 came from Queensland (most via local transmission), 72 from NSW, 15 from NT, 12 from SA, 8 from VIC, and 28 from WA. Notifications from New South Wales, South Australia, Victoria and Western Australia exceeded the five-year mean in each jurisdiction suggesting that the frequency of dengue is increasing [32].

Understanding the relationship between climate and dengue transmission is difficult because non-linear relationships exist between the daily survival of *Ae. aegypti*, the extrinsic incubation period (EIP) of the virus, temperature and humidity [33]–[35]. Forecasted regional warming in Australia may lengthen and intensify the dengue transmission season by shortening the mosquitoes' EIP, although it is important to note that dengue epidemics appear to be more strongly influenced by intrinsic population dynamic (epidemiological) processes than by climate [36]. Even so, any temporal extension effect in the transmission season will follow the expansion of potential larval sites that is now underway in Australia. Thus, while the issue of regional warming is important, the expansion of large rainwater tanks throughout urban regions of Australia is at present a prevailing human adaptation with more immediate possibilities for changing vector distributions in Australia than the direct warming effects projected by anthropogenic climate change scenarios. Whether southern Australia's current drought is due to the region's natural climate variability or part of a changing climate pattern, will continue to be debated by some. Nonetheless, it is important to avoid the cycle where human changes in water storage result in an *Ae. aegypti* range expansion followed by dengue epidemics seeded by viremic travellers [4],[37]. Additionally, domestic water storage can sustain *Ae. aegypti* populations (and dengue transmission) in regions not normally suitable for its survival [38], while active government and community contributions can remove established *Ae. aegypti* populations (and dengue) from areas where it has been endemic [39] – and both of these are human modifications.

In Australia, ineffectively screened domestic rainwater tanks have been identified as key containers with respect to *Ae. aegypti* productivity [40],[41]. The introduction of reticulated water systems in towns and cities throughout Australia is believed responsible for a major range contraction of *Ae. aegypti* over the last 50 years. This trend may now be reversed as humans adapt to climate-change-induced drought conditions – the increased use of domestic water storage in tanks could deliver stable primary larval sites into urban neighbourhoods. In Queensland's capital city, Brisbane – which is currently *Ae. aegypti* free – severe water shortages resulted in escalating water restrictions with an eventual prohibition on the use of all outside reticulated water outlets (November 2007–July 2008) and 75,000 domestic water tanks being installed by late 2007. This number of tanks represents approximately 21% of households with reticulated water in the Brisbane area (F. Chandler, Brisbane City Council, pers. comm.). Additionally, ad hoc uncontrolled water tanks are now also commonly being used to store rainwater, adding to the potential surfeit of stable breeding sites around Australia that are likely to facilitate the expansion risk of *Ae. aegypti* into urban areas. It is unlikely that any of these water storage tanks – government approved or not – will be maintained sufficiently to prevent mosquito access in the long term.

The flight range for *Ae. aegypti* is understood to be generally small: mark-release recapture experiments show them to have a flight range of only hundreds of metres [42]–[44]. However, these estimates are limited in time and space, being derived from a snapshot of one or a few gonotrophic cycles which take place in the context of an abundance of ovipositing sites. Longer distance flight range dispersal may be more common, especially when ovipositing sites are rare, but this is difficult to quantify [45],[46]. Human mediated long distance dispersal events are mostly responsible for *Ae. aegypti* movement: their highly domestic nature and desiccation-resistant eggs facilitate successful movement via human transport routes. Surveys in Queensland in the 1990s [17] and 1990–2005 (P. Mottram, unpublished) reveal *Ae. aegypti* collections from over 70 townships and this number is likely an underestimate. As the numbers of individuals and populations of *Ae. aegypti* increase in Queensland towns, the incursion risk beyond these regions via human-induced long distance dispersal events also increases, and with the presence of new stable oviposition sites growing, the expansion of this dengue vector must now be expected.

Operations to remove *Ae. aegypti* incursions are resource-heavy, often requiring both government legislation and widespread community cooperation to reduce adult mosquito populations. A recent example from a 2004 incursion of *Ae. aegypti* into the small Northern Territory town of Tennant Creek (pop 3,200) from Queensland resulted in a two-year eradication campaign that required 11 personnel and cost approximately $1.5 million and was achieved in 2006 [8].

### Conclusion

Determining the potential distribution of *Ae. aegypti* in Australia using climatic parameters can be problematic and in this case produced results that neither fully match the known distribution, nor reveal cold climate limits in Australia. Reasons for this may exist in the difficulty of relating the point occurrence data of a species' distribution that is closely tied to humans – unlike native mosquito species in Australia where GARP models appear more representative of known distributions [26],[47]. We must also consider the limited climatic parameters available through the OzClim climate scenario generator that reduced the GARP modelling to a subset of environmental parameters that may have little influence on the organism. Because the GARP models showed no cold temperature limits for *Ae. aegypti* in Australia, we also assessed two published theoretical cold temperature limits across Australia. These temperature limit projections also could not contain all collection sites, which may suggest that in Australia, climate - and in particular temperature - plays a less important role in determining the range of this species due to a combination of its intimate relationship with humans and our propensity to store water. This is where the use of statistical approaches and point occurrence data to evaluate species' distribution may be weak and integrating life processes parameters such as the effects of climate on reproduction and larval development may be more practical and informative.

If it is an assumption that burgeoning domestic water tanks will provide stable larval sites for *Ae. aegypti*, then the synthesis of our GARP modelling, the theoretical climate limits and the historical distribution of this mosquito strongly suggest that a distributional expansion is possible and could expose the majority of Australia's population to this dengue vector. Additionally, viewing this synthesis of *Ae. aegypti* in Australia with dengue transmission climate limits obtained from historical Australian dengue epidemics suggests a real risk of dengue transmission occurring in regions ranging well beyond north Queensland during the summer months.

We conclude that if the installation and maintenance of domestic water storage tanks is not tightly controlled today, *Ae. aegypti* could be spread by humans to cohabit with the majority of Australia's population, presenting a high potential dengue transmission risk during our warm summers.

## Supporting Information

Table S1 *Aedes aegypti* collection sites in Australia.
(0.69 MB RTF)
