# Supplementary material for: Australia's Dengue Risk Driven by Human Adaptation to Climate Change
Source: PLoS Negl Trop Dis. 2009 May 5;3(5):e429. doi: 10.1371/journal.pntd.0000429 (PMC2671609; doi:10.1371/journal.pntd.0000429)
Supplement: Table S1 — Aedes aegypti collection sites in Australia. (0.69 MB RTF) [file pntd.0000429.s001.rtf]

Supplementary Table 1. Collection sites of Ae. aegypti in Australia

UIN	Location	State	Longitude	Latitude	Source	
1	Ayr	Q	147.4	-19.566667	Sinclair (1992)	
2	Badu Island	TS	142.15	-10.116667	Sinclair (1992)	
3	Boulia	Q	139.9	-22.9	Sinclair (1992)	
4	Bowen	Q	148.233333	-20	Sinclair (1992)	
5	Cairns	Q	145.766667	-16.916667	Sinclair (1992)	
7	Cardwell	Q	146.016667	-18.25	Sinclair (1992)	
8	Charters Towers	Q	146.25	-20.066667	Sinclair (1992)	
10	Cloncurry	Q	140.5	-20.7	Sinclair (1992)	
11	Coconut Island	TS	143.05	-10.05	Sinclair (1992)	
12	Collinsville	Q	147.833333	-20.55	Sinclair (1992)	
13	Darnley Island	TS	143.75	-9.583333	Sinclair (1992)	
14	Dauan Island	TS	142.533333	-9.4	Sinclair (1992)	
15	Duaringa	Q	149.666667	-23.7	Sinclair (1992)	
16	Emerald	Q	148.15	-23.516667	Sinclair (1992)	
17	Georgetown	Q	143.533333	-18.283333	Sinclair (1992)	
18	Gladstone	Q	151.25	-23.833333	Sinclair (1992)	
19	Gordonvale	Q	145.783333	-17.083333	Sinclair (1992)	
20	Hammond Island	TS	142.216667	-10.55	Sinclair (1992)	
21	Home Hill	Q	147.4	-19.65	Sinclair (1992)	
22	Horn Island	TS	142.283333	-10.6	Sinclair (1992)	
23	Hughenden	Q	144.183333	-20.833333	Sinclair (1992)	
24	Ingham	Q	146.15	-18.65	Sinclair (1992)	
25	Innisfail	Q	146.016667	-17.516667	Sinclair (1992)	
26	Julia Creek	Q	141.733333	-20.65	Sinclair (1992)	
27	Mabuiag Island	TS	142.183333	-9.95	Sinclair (1992)	
28	Mackay	Q	149.183333	-21.133333	Sinclair (1992)	
29	Magnetic Island	Q	146.816667	-19.133333	Sinclair (1992)	
30	Marlborough	Q	149.883333	-22.8	Sinclair (1992)	
31	Mareeba	Q	145.416667	-16.983333	Sinclair (1992)	
32	Mingela	Q	146.633333	-19.866667	Sinclair (1992)	
33	Mission Beach	Q	146.1	-17.866667	Sinclair (1992)	
34	Moa Island	TS	142.266667	-10.183333	Sinclair (1992)	
35	Mossman	Q	145.366667	-16.45	Sinclair (1992)	
36	Mount Isa	Q	139.483333	-20.716667	Sinclair (1992)	
38	Murray Island	TS	144.05	-9.9	Sinclair (1992)	
39	Newell	Q	145.4	-16.416667	Sinclair (1992)	
40	Pentland	Q	145.4	-20.516667	Sinclair (1992)	
41	Port Douglas	Q	145.45	-16.033333	Sinclair (1992)	
42	Prince of Wales Island	TS	142.183333	-10.683333	Sinclair (1992)	
43	Proserpine	Q	148.566667	-20.4	Sinclair (1992)	
44	Ravenswood	Q	146.883333	-20.083333	Sinclair (1992)	
45	Richmond	Q	143.133333	-20.716667	Sinclair (1992)	
46	Rockhampton	Q	150.5	-23.366667	Sinclair (1992)	
48	Saibai Island	TS	142.683333	-9.4	Sinclair (1992)	
49	Sarina Beach	Q	149.3	-21.383333	Sinclair (1992)	
50	Sue Island	TS	142.816667	-10.2	Sinclair (1992)	
51	Thursday Island	TS	142.216667	-10.583333	Sinclair (1992)	
52	Townsville	Q	146.816667	-19.25	Sinclair (1992)	
53	Tully	Q	145.916667	-17.916667	Sinclair (1992)	
54	Winton	Q	143.033333	-22.383333	Sinclair (1992)	
55	Yam Island	TS	142.766667	-9.9	Sinclair (1992)	
56	Yorke Island	TS	143.433333	-9.733333	Sinclair (1992)	
57	Alpha	Q	146.633333	-23.65	P. Mottram	
58	Barcaldine	Q	145.283333	-23.55	P. Mottram	
59	Biloela	Q	150.5	-24.4	P. Mottram	
60	Blackall	Q	145.466667	-24.416667	P. Mottram	
61	Bluff	Q	149.066667	-23.566667	P. Mottram	
62	Capella	Q	148.02	-23.08	P. Mottram	
63	Charleville	Q	146.23	-26.4	P. Mottram	
64	Chinchilla	Q	150.62	-26.73	P. Mottram	
65	Clermont	Q	147.63	-22.82	P. Mottram	
68	Gayndah	Q	151.6	-25.62	P. Mottram	
70	Goomeri	Q	152.07	-26.19	P. Mottram	
71	Longreach	Q	144.24	-23.44	P. Mottram	
72	Monto	Q	151.12	-24.86	P. Mottram	
73	Mount Morgan	Q	150.39	-23.64	P. Mottram	
74	Rockhampton	Q	150.51	-23.37	P. Mottram	
75	Roma	Q	148.78	-26.57	P. Mottram	
76	Taroom	Q	149.79	-25.64	P. Mottram	
77	Yeppoon	Q	150.74	-23.12	P. Mottram	
78	Aberdeen	NSW	150.9	-32.166667	Ferguson (1927)	
79	Alstonville	NSW	153.433333	-28.816667	Ferguson (1927)	
80	Ardlethan	NSW	146.9	-34.333333	Russell et al. (1984)	
81	Ashford	NSW	151.083333	-29.3	Ferguson (1927)	
82	Ballimore	NSW	148.916667	-32.216667	Russell et al. (1984)	
83	Ballina	NSW	153.533333	-28.816667	Ferguson (1927)	
84	Bangalow	NSW	153.516667	-28.666667	O'Gower (1956)	
85	Barraba	NSW	150.616667	-30.366667	Ferguson (1927)	
86	Barringun	NSW	145.7	-29	Ferguson (1927)	
87	Bathurst	NSW	149.566667	-33.416667	O'Gower (1956)	
88	Bellingen	NSW	152.9	-30.433333	Ferguson (1927)	
89	Bethungra	NSW	147.85	-34.75	O'Gower (1956)	
90	Bingara	NSW	150.566667	-29.85	Ferguson (1927)	
91	Binnaway	NSW	149.383333	-31.55	Ferguson (1927)	
92	Blayney	NSW	149.25	-33.516667	O'Gower (1956)	
93	Bob's Farm	NSW	152.016667	-32.75	O'Gower (1956)	
94	Boggabri	NSW	150.033333	-30.7	Ferguson (1927)	
95	Boolaroo	NSW	151.616667	-32.933333	Ferguson (1927)	
96	Bourke	NSW	145.95	-30.066667	Ferguson (1927)	
97	Bowraville	NSW	152.85	-30.616667	Ferguson (1927)	
98	Breadalbane	NSW	149.433333	-34.783333	O'Gower (1956)	
99	Brewarrina	NSW	146.866667	-29.933333	Ferguson (1927)	
100	Brooklyn	NSW	151.216667	-33.533333	O'Gower (1956)	
101	Brunswick Heads	NSW	153.55	-28.516667	O'Gower (1956)	
102	Bulahdelah	NSW	152.183333	-32.383333	Ferguson (1927)	
103	Bundarra	NSW	150.883333	-30.2	Ferguson (1927)	
104	Byron Bay	NSW	153.616667	-28.633333	Ferguson (1927)	
105	Callubri	NSW	147.283333	-31.916667	O'Gower (1956)	
106	Camden	NSW	150.683333	-34.033333	O'Gower (1956)	
107	Canowindra	NSW	148.683333	-33.566667	O'Gower (1956)	
108	Carcoar	NSW	149.133333	-33.583333	O'Gower (1956)	
109	Casino	NSW	153.05	-28.833333	O'Gower (1956)	
110	Coffs Harbour	NSW	153.116667	-30.3	Ferguson (1927)	
111	Collarendabri	NSW	148.566667	-29.533333	Ferguson (1927)	
112	Cootamundra	NSW	148.033333	-34.633333	O'Gower (1956)	
113	Coraki	NSW	153.283333	-28.983333	Ferguson (1927)	
114	Cowra	NSW	148.65	-33.816667	Russell et al. (1984)	
115	Cudgegong	NSW	149.816667	-32.783333	O'Gower (1956)	
116	Culcairn	NSW	147.033333	-35.65	O'Gower (1956)	
117	Delungra	NSW	150.8	-29.633333	Ferguson (1927)	
118	Denman	NSW	150.683333	-32.366667	Ferguson (1927)	
119	Dora Creek	NSW	151.5	-33.066667	Russell et al. (1984)	
120	Drake	NSW	152.366667	-28.9	Ferguson (1927)	
121	Dubbo	NSW	148.6	-32.233333	O'Gower (1956)	
122	Dunedoo	NSW	149.033333	-32	O'Gower (1956)	
123	Frederickton	NSW	152.866667	-31.016667	Russell et al. (1984)	
124	Geurie	NSW	148.5	-32.383333	O'Gower (1956)	
125	Goolma	NSW	149.266667	-32.366667	O'Gower (1956)	
126	Gosford	NSW	151.333333	-33.416667	O'Gower (1956)	
127	Grafton	NSW	152.933333	-29.666667	Ferguson (1927)	
128	Greta	NSW	151.383333	-32.666667	Ferguson (1927)	
129	Gulgong	NSW	149.533333	-32.35	O'Gower (1956)	
130	Gunnedah	NSW	150.25	-30.966667	Ferguson (1927)	
131	Harden	NSW	148.366667	-34.533333	O'Gower (1956)	
132	Inverell	NSW	151.116667	-29.766667	Ferguson (1927)	
133	Junee	NSW	147.566667	-34.866667	O'Gower (1956)	
134	Kellyville	NSW	150.95	-33.716667	O'Gower (1956)	
135	Kempsey	NSW	152.833333	-31.066667	Ferguson (1927)	
136	Kingswood	NSW	150.9	-31.15	O'Gower (1956)	
137	Kyogle	NSW	153	-28.616667	Ferguson (1927)	
138	Lawrence	NSW	153.1	-29.483333	Ferguson (1927)	
139	Lismore	NSW	153.283333	-28.8	Ferguson (1927)	
140	Liverpool	NSW	150.916667	-33.916667	O'Gower (1956)	
141	Lyndhurst	NSW	149.033333	-33.65	O'Gower (1956)	
142	Macksville	NSW	152.916667	-30.716667	Ferguson (1927)	
143	Maclean	NSW	153.2	-29.45	Ferguson (1927)	
144	Maitland	NSW	151.55	-32.716667	Ferguson (1927)	
145	Manilla	NSW	150.716667	-30.733333	Ferguson (1927)	
146	Merriwa	NSW	150.35	-32.116667	Ferguson (1927)	
147	Merriwagga	NSW	145.616667	-33.8	O'Gower (1956)	
148	Merrygoen	NSW	149.233333	-31.8	O'Gower (1956)	
149	Moree	NSW	149.833333	-29.45	Ferguson (1927)	
150	Morisset	NSW	151.5	-33.1	O'Gower (1956)	
151	Morpeth	NSW	151.616667	-32.716667	Ferguson (1927)	
152	Mudgee	NSW	149.583333	-32.566667	O'Gower (1956)	
153	Mullumbimby	NSW	153.5	-28.533333	Russell et al. (1984)	
154	Mumbil	NSW	149.05	-32.7	O'Gower (1956)	
155	Mummulgum	NSW	152.8	-28.866667	Russell et al. (1984)	
156	Murrurundi	NSW	150.833333	-31.75	Ferguson (1927)	
157	Murwillumbah	NSW	153.383333	-28.316667	Ferguson (1927)	
158	Muswellbrook	NSW	150.883333	-32.25	Ferguson (1927)	
159	Narrabri	NSW	149.766667	-30.316667	Ferguson (1927)	
160	Narrandera	NSW	146.55	-34.733333	O'Gower (1956)	
161	Nelson Bay	NSW	152.15	-32.7	O'Gower (1956)	
162	Newcastle	NSW	151.783333	-32.916667	O'Gower (1956)	
163	Nyngan	NSW	147.166667	-31.533333	O'Gower (1956)	
164	Ourimbah	NSW	151.366667	-33.35	O'Gower (1956)	
165	Parkes	NSW	148.25	-33.083333	O'Gower (1956)	
166	Penrith	NSW	150.75	-33.733333	O'Gower (1956)	
167	Pilliga	NSW	148.9	-30.35	Ferguson (1927)	
168	Port Stephens	NSW	152.066667	-32.683333	Ferguson (1927)	
169	Quandialla	NSW	147.783333	-34	O'Gower (1956)	
170	Quirindi	NSW	150.683333	-31.483333	Ferguson (1927)	
171	Rylstone	NSW	149.966667	-32.783333	O'Gower (1956)	
172	St Albans	NSW	150.983333	-33.283333	O'Gower (1956)	
173	Scone	NSW	150.866667	-32.033333	Ferguson (1927)	
174	Singleton	NSW	151.166667	-32.55	Ferguson (1927)	
175	Smithtown	NSW	152.933333	-31	Russell et al. (1984)	
176	Sofala	NSW	149.683333	-33.016667	O'Gower (1956)	
177	Stockinbingal	NSW	147.883333	-34.483333	O'Gower (1956)	
178	Stockton	NSW	151.783333	-32.9	Ferguson (1927)	
179	Tabulam	NSW	152.516667	-28.85	Ferguson (1927)	
180	Tamworth	NSW	150.916667	-31.083333	Ferguson (1927)	
181	Taree	NSW	152.45	-31.9	Ferguson (1927)	
182	Temora	NSW	147.533333	-34.433333	O'Gower (1956)	
183	Tenterfield	NSW	152.016667	-29.033333	Ferguson (1927)	
184	Tweed Heads	NSW	153.533333	-28.166667	Ferguson (1927)	
185	Tyalgum	NSW	153.216667	-28.35	O'Gower (1956)	
186	Ulmarra	NSW	153.033333	-29.616667	Ferguson (1927)	
187	Wagga Wagga	NSW	147.366667	-35.1	O'Gower (1956)	
188	Walgett	NSW	148.1	-30.016667	Ferguson (1927)	
189	Wallendbeen	NSW	148.166667	-34.516667	O'Gower (1956)	
190	Waratah	NSW	151.716667	-32.883333	Ferguson (1927)	
191	Wardell	NSW	153.466667	-28.933333	Ferguson (1927)	
192	Warialda	NSW	150.516667	-29.4	Ferguson (1927)	
193	Wauchope	NSW	152.733333	-31.45	Ferguson (1927)	
194	Wee Waa	NSW	149.433333	-30.2	Ferguson (1927)	
195	Wellington	NSW	148.933333	-32.533333	O'Gower (1956)	
196	Werris Creek	NSW	150.65	-31.333333	Ferguson (1927)	
197	Windsor	NSW	150.8	-33.6	O'Gower (1956)	
198	Wingham	NSW	152.366667	-31.85	Ferguson (1927)	
199	Woy Woy	NSW	151.316667	-33.483333	Russell et al. (1984)	
200	Wyong	NSW	151.416667	-33.266667	Ferguson (1927)	
201	Dirranbandi	Q	148.22	-28.58	(Kay et al., 1984)	
202	Darwin	NT	130.84	-12.46	O'Gower (1956)	
203	Adelaide R	NT	131.1	-13.23	O'Gower (1956)	
204	Brocks Ck	NT	131.41	-13.45	O'Gower (1956)	
205	Daly Waters	NT	133.36	-16.26	O'Gower (1956)	
206	Katherine	NT	132.26	-14.46	O'Gower (1956)	
207	Koolpinya	NT	131.26	-12.62	O'Gower (1956)	
208	Newcastle W	NT	133.4	-17.37	O'Gower (1956)	
209	Pine Ck	NT	131.83	-13.82	O'Gower (1956)	
210	Charles Pt	NT	130.62	-12.39	O'Gower (1956)	
211	Anthony Lag	NT	135.53	-17.97	O'Gower (1956)	
212	Roper R	NT	134.73	-14.73	O'Gower (1956)	
213	Beagle Bay	WA	122.66	-16.97	O'Gower (1956)	
214	Broome	WA	122.23	-17.96	O'Gower (1956)	
215	Derby	WA	123.62	-17.3	O'Gower (1956)	
216	Pt Hedland	WA	118.6	-20.31	O'Gower (1956)	
217	Yeeda	WA	123.64	-17.61	O'Gower (1956)	
218	Freemantle	WA	115.74	-32.05	(Lumley, Taylor, 1943)	
219	Tennant Ck	NT	134.23	-19.55	P.I. Whelan	
220	Groote Eyl	NT	136.45	-13.97	P.I. Whelan	
221	Camooweal	Q	138.12	-19.92	P.I. Whelan	
222	Beverley	WA	116.92	-32.1	Liehne (1991)	
223	Geraldton	WA	114.61	-28.77	Liehne (1991)	
224	Halls Ck	WA	127.66	-18.22	Liehne (1991)	
225	Harvey	WA	115.89	-33.07	Liehne (1991)	
226	Meekatharra	WA	118.49	-26.59	Liehne (1991)	
227	Moora	WA	116.01	-30.63	Liehne (1991)	
228	Wyndham	WA	128.12	-15.48	Liehne (1991)	
229	Karumba	Q	140.87	-17.48	(Kay et al., 1984)	
230	Mornington Is	Q	139.4	-16.55	(Kay et al., 1984)	
231	Normanton	Q	141.07	-17.67	(Kay et al., 1984)	
232	Cunnamulla	Q	145.68	-28.07	(Lumley, Taylor, 1943)	
233	Toowoomba	Q	151.95	-27.55	(Lumley, Taylor, 1943)	
234	Brisbane	Q	153.02	-27.46	(Lumley, Taylor, 1943)	
Q= Queensland; NSW= New South Wales; SA= South Australia; WA= Western Australia; NT= Northern Australia; TS= Torres Strait (far north Queensland)
